# Musculoskeletal Pain Among University Students and Its Correlations with Risk Factors: A Cross-Sectional Study

**DOI:** 10.3390/jcm14176076

**Published:** 2025-08-28

**Authors:** Sultan Ayyadah Alanazi, Faizan Zaffar Kashoo

**Affiliations:** Department of Physical Therapy and Health Rehabilitation, College of Applied Medical Sciences, Majmaah University, Al Majmaah 11952, Saudi Arabia; f.kashoo@mu.edu.sa

**Keywords:** musculoskeletal pain, risk factors, students, universities, stress, psychological, Saudi Arabia

## Abstract

**Background:** Several studies have examined the prevalence of musculoskeletal pain (MSP) among university students internationally. We aimed to assess the prevalence, pattern, and potential risk factors for MSP among Majmaah University students in Saudi Arabia. **Methods:** A cross-sectional questionnaire was administered to students from different faculties at Majmaah University. We collected data via the validated Arabic versions of the Standardized Nordic Musculoskeletal Questionnaire, the International Physical Activity Questionnaire, and the Perceived Stress Scale. Bivariate and multivariate logistic regression analyses were performed to explore associations between MSP and demographic, ergonomic, lifestyle, and psychosocial variables. **Results:** A total of 257 students (*n* = 195, 75.9% female; *n* = 62, 24.1% male) were included in this study. The 12-month prevalence of MSP was 225 (87.5%), with the lower back (*n* = 119, 46.3%) and neck (*n* = 113, 44.0%) regions being the most affected. Compared with male students, female students reported a higher MSP prevalence (90.3% vs. 79.0%, *p* = 0.035). The multivariable model was significant (likelihood-ratio χ^2^ = 26.042, df = 7, *p* < 0.001), accounted for Nagelkerke R^2^ = 0.182 of variance, and showed good calibration (Hosmer–Lemeshow χ^2^ = 8.505, df = 8, *p* = 0.386). Perceived stress was the only independent predictor of 12-month MSP (β = 0.084, adjusted OR = 1.087, 95% CI 1.034–1.143, Wald χ^2^ = 10.732, *p* = 0.001), while sex, smoking, academic workload, and sleep duration were non-significant (all *p* > 0.127). **Conclusions:** MSP appears to be prevalent among Majmaah University students, with psychological stress emerging as a key independent risk factor. Preventive strategies should include stress management prioritization and ergonomic and physical activity education to support university student well-being.

## 1. Introduction

Musculoskeletal pain (MSP) is a significant and growing global public health concern, particularly among young adults [1,2]. MSP, commonly leading to chronic pain, discomfort, or functional impairment, can result from any injury or trauma to the musculoskeletal structures (bones, joints, or soft tissues) and can be diagnosed clinically or reported by individuals [3,4]. MSP can reduce an individual’s quality of life, physical functioning, and academic performance, particularly among university students [5,6].

The transition to university life is often associated with drastic lifestyle and behavioral changes, including reduced physical activity, prolonged sedentary behavior, irregular sleep schedules, and increased psychological stress [7,8]. University students are commonly exposed to risk factors such as extended periods of sitting, suboptimal ergonomics in study environments, and excessive use of electronic devices [9]. These behaviors may contribute significantly to the development of MSP, specifically in the neck, shoulders, and lower back regions [5,7,10,11].

Research has revealed a high prevalence of MSP in student populations, with lifetime prevalence rates of neck and lower back pain ranging from 30% to 70% [10,12,13]. MSP is a common health concern among university students in Saudi Arabia, with reported prevalence rates ranging from 64.8% to over 70% [5,11,14]. However, research focusing on MSP and associated risk factors among university students in Saudi Arabia remains limited. If left unmanaged, MSP can have implications beyond the associated symptoms (pain and discomfort), such as absenteeism, reduced academic performance, and long-term physical and psychological consequences [5,15].

Despite a growing body of literature on MSP in college students, existing surveys typically focus on isolated dimensions such as posture or activity level without integrating the full spectrum of behavioral, biomechanical, and psychosocial determinants that shape students’ musculoskeletal health [16]. Keywords such as cumulative screen time, habitual sleep position, and perceived stress are often overlooked, resulting in a limited understanding of the underlying causes of the high prevalence of neck, shoulder, and lower back pain complaints among university populations.

Several standardized tools have been widely utilized to assess musculoskeletal health, physical activity, and stress levels. The Nordic Musculoskeletal Questionnaire (NMQ) [17], the International Physical Activity Questionnaire (IPAQ-Arabic) [18], and the Perceived Stress Scale (PSS-10) [19] quantify MSP, physical activity, and stress, respectively. Given the high prevalence of MSP among university students and the limited integrative evidence from Saudi Arabia, it is important to examine not only lifestyle and ergonomic factors but also psychosocial determinants such as stress. We hypothesized that psychological stress would emerge as a stronger independent predictor of MSP compared with traditional ergonomic or behavioral risk factors. Accordingly, this study aimed to determine the prevalence and distribution of MSP among university students and to explore its associations with demographic, ergonomic, lifestyle, and psychosocial risk factors.

## 2. Methods

This cross-sectional study was conducted across all faculties at Majmaah University, Saudi Arabia, and was reported in accordance with the Strengthening the Reporting of Observational Studies in Epidemiology (STROBE) Statement checklist for cross-sectional studies [20]. The study was ethically approved by the Institutional Review Board (IRB) at Majmaah University (IRB approval No.: MUREC-Sep.18/COM-2024/44), and all procedures involving human participants were carried out in accordance with the ethical standards of the 1964 Declaration of Helsinki and its later amendments. All participants provided informed consent before participation.

### 2.1. Participants

The sample comprised students (bachelor’s and master’s) aged ≥18 years. The inclusion criteria were as follows: (1) full-time enrollment at Majmaah University and (2) voluntary agreement to participate through informed consent. Students who reported chronic musculoskeletal disorders unrelated to lifestyle factors (e.g., congenital abnormalities and posttraumatic injuries) were excluded to minimize confounding. We targeted a diverse student population representative of various academic disciplines and years of study at Majmaah University. Data were collected between September 2024 and May 2025. Students were invited to participate through official university-wide announcements (e.g., online platforms). Participation was voluntary and anonymous, and no identifying data were collected, thereby eliminating potential academic or grading conflicts of interest.

### 2.2. Sample Size

The Qualtrics Sample Size Calculator indicated that a population size of 20,000 with a 90% confidence level and a 6% margin of error yields an ideal sample size of 187 participants. A final target sample of 250 students was established to account for potential nonresponse and incomplete surveys. Our sampling method was stratified convenience sampling: we divided the student population into 11 strata based on college, then sent participation invitations to all eligible students within each stratum. While this approach ensured proportional representation, participant selection within strata was not random.

### 2.3. Data Collection Questionnaire

We used an online questionnaire specifically designed to collect data for this study. The questionnaire contained five sections, each intended to capture different dimensions relevant to musculoskeletal health among university students. The first section collected demographic information, including age, sex, academic year, study level (bachelor’s or master’s degree), height, and weight. The body mass index (BMI) was calculated via the World Health Organization’s standard classification system to evaluate potential weight-related risk factors. Perceived academic workload was assessed via a single self-report item asking students to rate the typical intensity of their academic commitments. Participants chose from four categories: low, moderate, high, or very high workload. This item was intended to capture the students’ general sense of how demanding their studies were (in terms of course load, assignments, and study pressure).

The second section focused on musculoskeletal symptoms. The Arabic version of the NMQ was utilized to assess the presence, location, and duration of pain [17]. This widely validated tool allows the identification of self-reported musculoskeletal discomfort in nine anatomical regions over the preceding 12 months and 7 days. The participants were also asked to report whether these symptoms interfered with their daily or academic activities.

The third section of the study included the short version of the IPAQ-Arabic [18]. The IPAQ-Arabic has been translated and culturally adapted to ensure relevance and accuracy for the Arabic-speaking population and is commonly used for measuring physical activity across various populations.

The fourth section assessed psychosocial factors via the 10-item PSS-10, a validated scale designed to evaluate the degree to which individuals perceive situations in their lives as stressful [19]. We included the PSS-10 scale to explore potential correlations between psychological stress and the occurrence of MSP.

In addition to the inclusion of validated domains captured by the NMQ (symptom location), the IPAQ (physical-activity load), and the PSS (psychological stress), we deliberately expanded the questionnaire to cover a wide range of emergent or underrepresented determinants highlighted by recent epidemiological evidence [16]. In the last section, the participants were questioned about (i) cumulative smartphone use and desktop/laptop use (screen-time dose), (ii) sleep hygiene (average nightly hours) and usual bedtime and habitual sleeping posture, (iii) anthropometrics for body mass index calculations, (iv) tobacco smoking status, (v) self-reported chronic musculoskeletal or systemic disease, and (vi) their academic context (college affiliation and year of study as proxies for discipline-specific ergonomic loads and workload peaks). Adding the previous items to the validated instruments ensured that the final questionnaire captured well-substantiated and practice-relevant risk factors that were missing from existing musculoskeletal surveys, offering a more comprehensive picture of musculoskeletal vulnerability among university students.

The final questionnaire was pilot-tested with 30 students before the main study to ensure it was clear and reliable. Feedback from the pilot led to minor modifications to improve the clarity of the language and layout. We then finalized the questionnaire and distributed it via university communication channels.

### 2.4. Outcome Definition

Twelve-month MSP was assessed using the Arabic version of the NMQ, which screens for pain in nine anatomical regions: neck, shoulders, elbows, wrists/hands, upper back, lower back, hips/thighs, knees, and ankles/feet. Participants were asked whether they had experienced pain, aching, or discomfort in any of these regions at any time during the past 12 months. A response of “yes” in one or more regions was considered a positive screen for 12-month MSP.

To restrict the case definition to clinically meaningful problems, a chronicity filter was then applied in line with ICD-11: only those whose symptoms occurred on at least one day per week for ≥12 consecutive weeks within that 12-month window were classified as MSP “cases”. Respondents who reported no pain in the past year or pain episodes of shorter duration were coded as the reference (non-MSP) group.

#### Scoring and Cut–Off Score for Questionnaires

All the ten PSS items were first coded 0 = “No, never”, 1 = “Rarely”, 2 = “Sometimes”, 3 = “Mostly”, 4 = “Always/Permanent”, preserving the original 0–4 metric. In the standard PSS, the four positively worded items (4, 5, 7, 8) are reverse-scored (0↔4, 1↔3); however, our adapted questionnaire phrased every item negatively, so no reversal was required. The ten numeric responses were then summed to yield a total score ranging from 0 to 40, with higher values indicating greater perceived stress. For descriptive purposes, we classified totals of 0–13 as low, 14–26 as moderate, and 27–40 as high perceived stress, following widely used cut-points derived from the original PSS manual.

Metabolic equivalent of task (MET) is a physiological unit that expresses the energy cost of physical activities as a multiple of the resting metabolic rate. MET is defined as the amount of oxygen consumed while sitting quietly—approximately 3.5 mL O_2_·kg^−1^·min^−1^ (≈1 kcal·kg^−1^·h^−1^) [21]. Thus, an activity valued at 4 METs requires roughly four times the energy expenditure of resting. Responses to the four categorical IPAQ items were converted to minutes using established mid-points: sitting per day: <1 h = 45 min, 1–2 h = 90 min, 3–4 h = 210 min, 5–6 h = 330 min, >6 h = 420 min; walking per day: <10 min = 0 min, 10–30 min = 20 min, 30–60 min = 45 min, >60 min = 90 min; moderate- or vigorous-intensity activity per week: <30 min = 15 min, 30–60 min = 45 min, 60–120 min = 90 min, >120 min = 150 min. Walking minutes were multiplied by 3.3 METs and by seven days, whereas moderate and vigorous minutes were multiplied by 4.0 METs and 8.0 METs, respectively, with each domain truncated at 180 min in accordance with the IPAQ data-processing manual [22]. The three domain scores were summed to yield a total MET-minutes·week^−1^ value. Participants were classified as low (<600 MET-min·wk^−1^), moderate (600–2999) or high/HEPA-active (≥3000 or vigorous activity ≥ 1500 MET-min·wk^−1^) following WHO guidelines [23].

### 2.5. Statistical Analysis

Chi-square and independent-samples *t* tests were used to assess bivariate associations between MSP and categorical (e.g., sex and physical activity) or continuous (e.g., stress and BMI) variables. A parsimonious multivariable logistic regression model (*n* = 257) was fitted to estimate independent associations with 12-month MSP, retaining only conceptually upstream predictors selected through the Hosmer–Lemeshow purposeful-selection procedure—sex, perceived academic load, current smoking status, average sleep duration, and PSS score—thereby satisfying the ≥10-events-per-variable recommendation and avoiding over-adjustment bias.

Continuous variables were checked for normality. The stress scores were approximately normally distributed (Shapiro–Wilk W = 0.988, *p* = 0.038), with slight skewness; BMI was right-skewed due to a high outlier (W = 0.859, *p* < 0.001; BMI ≈ 62), which was retained. The sensitivity analysis excluding the outliers revealed a negligible impact (BMI remained nonsignificant: *p* = 0.56). No extreme outliers were found for stress or screen time. The homogeneity of variances was met (e.g., stress by MSP status: Levene’s F (1, 255) = 1.21, *p* = 0.27). Given the sample size, parametric tests were considered robust. Analyses were conducted via SPSS version 20 (IBM, Armonk, NY, USA).

## 3. Results

### 3.1. Participant Characteristics

A total of 257 students (*n* = 195, 75.9% female; *n* = 62, 24.1% male) were included in the study. Most of the participants were in their first or second academic year (*n* = 63, 49.4% in Years 1–2), with ages ranging primarily from 18 to 25 years. The participants’ mean weight and height were 64.2 kg and 163.4 cm, respectively, yielding a mean BMI of 23.8 (SD = 5.7, range 15.4–62.4).

On average, the students reported high screen time and used smartphones and computers for a combined period of ~10.7 h per day (ranging up to 20 h). Almost half of the participants (*n* = 124, 48.2%) reported no regular physical activity, whereas the remainder frequently engaged in aerobic exercise (*n* = 76, 29.5%), resistance training (*n* = 35, 13.6%), or stretching/yoga (*n* = 22, 8.5%). The mean perceived stress score was 19.1 (SD = 8.9) on a 0–40 scale, indicating moderate average stress levels.

### 3.2. Prevalence of MSP

Overall, 225 students (87.5%) reported experiencing MSP in at least one body region during the past 12 months (12-month prevalence). In the preceding 7 days, 57 students (22.2%) reported MSP (point prevalence). Approximately 94 students (36.6%) indicated that their pain had limited their ability to perform routine activities (e.g., work or chores) at some point in the previous year. A summary of regional MSP data is provided in Table 1.

### 3.3. Bivariate Associations

Chi-square tests indicated significant associations for sex, smoking status, prior consultation for a chronic musculoskeletal disorder, sleep discomfort, and perceived stress level. Females reported pain more often than males, χ^2^(1) = 5.437, *p* = 0.020, with 90.3% (176/195) of females versus 79.0% (49/62) of males endorsing MSP. Smokers were less likely to report pain than non-smokers, χ^2^(1) = 4.171, *p* = 0.041; 72.2% (13/18) of smokers compared with 88.7% (212/239) of non-smokers reported pain. All students who had previously consulted a clinician for a chronic musculoskeletal condition reported pain (36/36), whereas 85.5% (189/221) of their peers without such a history did so, χ^2^(1) = 5.954, *p* = 0.015. Nocturnal discomfort that caused frequent position changes was also related to higher pain prevalence, χ^2^(1) = 5.793, *p* = 0.016 (92.9%, 26/28 vs. 86.4%, 158/183). Finally, perceived stress exhibited a graded relationship with pain, χ^2^(2) = 14.267, *p* = 0.001, increasing from 75.8% in the low-stress group to 98.1% in the high-stress group. No other variables reached statistical significance at the 0.05 level (Table 2).

### 3.4. Multivariate Logistic Regression

The multivariable logistic-regression model (likelihood-ratio χ^2^ = 26.042, df = 7, *p* < 0.001) demonstrated satisfactory explanatory power for the presence of MSP within the preceding 12 months, accounting for 18.2% of the variance (Nagelkerke R^2^ = 0.182) and showing good calibration (Hosmer–Lemeshow χ^2^ = 8.505, df = 8, *p* = 0.386). After simultaneous adjustment for sex, smoking status, academic workload, and hours of sleep, only perceived stress retained statistical significance: each one-point increment on the PSS-10 increased the odds of MSP by 8.7% (β = 0.084; Wald = 10.732; *p* = 0.001; adjusted OR = 1.087; 95% CI = 1.034–1.143), implying a 2.29-fold elevation in risk across a 10-point rise in stress. In contrast, being male was associated with a non-significant 40% reduction in MSP odds (OR = 0.597; 95% CI = 0.239–1.496; *p* = 0.271), current smoking conferred a non-significant 84% increase (OR = 1.843; 95% CI = 0.472–7.190; *p* = 0.379), and none of the academic-workload categories—low (OR = 0.462; *p* = 0.512), moderate (OR = 0.602; *p* = 0.642), or high (OR = 1.528; *p* = 0.733)—differed significantly from the reference (very high) group. Similarly, longer nightly sleep showed a protective trend that did not reach significance (OR = 0.615 per hour; 95% CI = 0.329–1.148; *p* = 0.127). Collectively, these results indicate that heightened perceived stress, rather than demographic, behavioral, or academic factors, is the principal independent determinant of MSP in this cohort (Table 3).

## 4. Discussion

This cross-sectional online survey of Majmaah University students revealed a high 12-month prevalence of MSP, most commonly affecting the lower back and neck (9 in 10 respondents). In the unadjusted analyses, female students and those reporting greater psychological stress were found to be more likely to report MSP; however, the multivariable logistic regression analysis identified perceived stress as the only independent predictor of MSP. Traditional risk indicators such as physical inactivity, prolonged screen time, poor study posture, higher BMI, and academic year were not significant after controlling for stress.

The findings answer the study’s primary question by documenting a substantial burden of MSP among Saudi university students. In contrast to expectations, ergonomic and lifestyle factors did not independently predict pain, suggesting that psychosocial stress may be a more salient mechanism in this relatively homogeneous cohort. These findings support the biopsychosocial model, indicating that physiological strain alone is insufficient to explain students’ pain; stress-related muscle tension, heightened pain sensitivity, or behavioral changes (e.g., reduced microbreaks during intense study) may mediate this relationship. This is consistent with biopsychosocial models of musculoskeletal disorders (MSDs), as well as occupational stress frameworks such as the job demand–control–support model and concepts of allostatic load, which posit that sustained stress responses create long-term physiological strain [24]. Our findings are supported by prior research showing that academic stress increases the risk of MSDs, with stressors related to life changes and academic pressure associated with 1.087-fold higher odds of MSDs [25]. Similar trends have been documented in other Middle Eastern university cohorts, where psychosocial stress was consistently a stronger determinant of musculoskeletal pain than ergonomic factors [26]. A previous cross-sectional study also revealed that emotional and physiological responses to stress significantly predict MSDs, particularly among females [25]. One plausible mechanism is that high perceived stress chronically activates the hypothalamic–pituitary–adrenal (HPA) axis and the sympathetic nervous system, leading to elevated cortisol and catecholamine levels [27]. This hormonal milieu increases resting muscle tone and promotes local ischemia and micro-trauma in musculoskeletal tissues [28]. At the same time, stress-induced release of pro-inflammatory cytokines and a drop in pain-inhibitory neurotransmitters lower the threshold for pain perception (central sensitization) [29]. Together with stress-related behaviors such as poor sleep, reduced physical activity, and maladaptive coping, these changes create a perfect storm that both initiates and exacerbates MSDs [30]. Sex differences may further compound these effects. Previous studies suggest that hormonal variations, differences in pain perception, and higher reported psychosocial stress among female students could partly explain the initially observed sex-based differences in MSP [31].

Prevalence estimates align with international reports of a 60–90% prevalence of MSP among undergraduates [10,32]. The dominance of lower back and neck pain is consistent with studies reporting data on South African and Canadian college students [7,33]. However, our null findings for physical inactivity contrast with meta-analytic evidence that sedentary behavior elevates MSP risk [12]. One explanation is that the high screen time and prolonged sitting habits observed across the participants could have reduced the differences between the groups. Thus, any potential effects that might have been detected between the groups could have been reduced or weakened because of these common behaviors. When everyone in a study spends much of their time sitting and using screens, the variations in outcomes or responses that could indicate different effects between groups may become less distinct or observable. Additionally, null associations may reflect measurement bias inherent to self-reported posture and screen use, which often lack granularity. Cross-sectional designs also preclude accounting for cumulative load or prolonged ergonomic strain, which are likely more relevant in older or occupational populations. Likewise, the non-significant BMI association differs from reports linking obesity to MSP [34]; however, our cohort’s mean BMI fell within the normal range, limiting direct comparisons. Consistent with previous research [35], stress emerged as a strong correlate, underscoring the increasing recognition of mental health drivers in MSP.

These data extend our knowledge by highlighting stress as a pivotal, modifiable risk factor in a Middle Eastern student population. Internationally, there is heterogeneity in the relative weighting of risk factors. While sedentary lifestyle has emerged as the predominant risk driver in East Asian cohorts [36], psychological stress predominates in European and Middle Eastern populations [37,38]. This variability suggests contextual, cultural, and methodological influences that shape the manifestation of MSP risk. Theoretically, these findings support models emphasizing central sensitization and psychoneuroendocrine pathways (stress response pathways) linking stress to MSP [39]. Practically, campus health services should integrate stress-management interventions (mindfulness programs and workload counseling) alongside ergonomic education to mitigate MSP. Given that ergonomic and activity variables were nonsignificant after adjustment, stand-alone posture or exercise campaigns may yield limited benefits unless psychological stress is concurrently addressed.

## 5. Limitations

This study has several limitations that should be considered. First, we used a cross-sectional design, which precludes causal inference; elevated stress may contribute to and result from MSP. MSP was self-reported, and we were unable to confirm diagnoses or investigate the underlying biomedical causes of pain. Thus, our results reflect symptom prevalence rather than clinically confirmed disorders. Second, self-reported measures can introduce recall and social desirability bias, particularly regarding physical activity and posture. Third, although the use of regression analysis strengthened the robustness of our findings, the relatively modest sample size drawn from a single institution limits the external generalizability of the results. The number of participants from some departments was small, limiting our ability to perform valid subgroup comparisons across academic disciplines. Future studies should aim to include larger, multi-center samples to enhance representativeness. Fourth, we did not measure certain potential confounders—for example, sleep quality, detailed ergonomic factors (e.g., chair height, monitor alignment), or objective physical activity tracking—and the absence of these data could have led to residual confounding, thereby masking some true associations between the risk factors we did examine and MSP outcomes. Fifth, one extreme BMI outlier slightly skewed the distributions despite robust analyses. Furthermore, we acknowledge that the NMQ does not quantify pain intensity, which is a limitation of our assessment tool. This means we could not analyze the severity of pain symptoms. However, our primary aim was to document the prevalence of MSP and its associations with risk factors (rather than the degree of pain), so the NMQ was appropriate for identifying whether pain was present in each body region. Importantly, the lack of intensity data does not affect our ability to identify pain cases or examine their correlates, although it limits any analysis of pain severity or intensity-related outcomes. Further, we did not collect information regarding analgesic or other medication use (type, dosage, timing), which may have influenced pain reporting. This omission is a limitation and should be addressed in future research.

## 6. Future Research Directions

Prospective cohort studies are needed to clarify the temporal relationships between stress and MSP and to test mediation pathways. Incorporating objective measures such as wearable activity trackers, posture sensors, and cortisol assays could reduce reporting bias and explain physiological mechanisms. Interventional trials combining stress-reduction strategies with ergonomic adjustments could determine their synergistic effects on MSP prevalence and severity. Expanding sampling to multiple universities, including diverse academic programs and balanced sex representations, will enhance external validity. Future research should also move beyond cross-sectional designs by employing longitudinal or interventional approaches, which would allow the establishment of causal relationships rather than mere associations. Finally, qualitative studies could explore students’ lived experiences of stress-related pain, informing culturally sensitive interventions.

## 7. Conclusions

This study reveals an alarmingly high prevalence of MSP among university students and identifies psychological stress—not traditional ergonomic or lifestyle factors—as the principal independent predictor. The findings underscore the need to move beyond purely biomechanical explanations and integrate mental health support within musculoskeletal health initiatives on campus. Addressing students’ stress may offer a key leverage point for reducing pain, improving well-being, and enhancing academic performance.

## Figures and Tables

**Table 1:** Prevalence of musculoskeletal pain by body region (past 12 months, 7 days, and activity limitation).

Body Region	Pain 12 m *n* (%)	Pain 7 d *n* (%)	Activity-Limiting *n* (%)
Neck	113 (44.0%)	19 (7.4%)	22 (8.6%)
Lower back (lumbar)	119 (46.3%)	10 (3.9%)	34 (13.2%)
Knees	35 (13.6%)	8 (3.1%)	12 (4.7%)
Wrists/hands	37 (14.4%)	9 (3.5%)	17 (6.6%)
Hips/thighs	40 (15.6%)	8 (3.1%)	11 (4.3%)
Ankles/feet	45 (17.5%)	14 (5.4%)	15 (5.8%)
Upper back	51 (19.8%)	6 (2.3%)	13 (5.1%)
Shoulder (left)	65 (25.3%)	13 (5.1%)	18 (7.0%)
Shoulder (right)	84 (32.7%)	19 (7.4%)	24 (9.3%)
Elbows (both)	8 (3.1%)	4 (1.6%)	2 (0.8%)

Note: *n* = 257 university students. Percentages use the full sample as the denominator and may not sum to 100% because respondents could report pain in multiple regions. “Pain 12 m” = any ache, discomfort, or trouble experienced at least once in the previous 12 months; “Pain 7 d” = symptoms during the week preceding the survey; “Activity-limiting” = pain that prevented normal work, study, or daily activities at any time in the past year.

**Table 2:** Bivariate association table.

Variable	Category	MSP-Yes *n* (%)	MSP-No *n* (%)	Pearson χ^2^(df), *p*
Age	18–20 y	94 (88.7%)	12 (11.3%)	4.956 (3), 0.175
	21–22 y	78 (91.8%)	7 (8.2%)	
	23–25 y	32 (82.1%)	7 (17.9%)	
	≥5 y	21 (77.8%)	6 (22.2%)	
Sex	Male	49 (79.0%)	13 (21.0%)	5.437 (1), 0.020
	Female	176 (90.3%)	19 (9.7%)	
College	Applied College	4 (80.0%)	1 (20.0%)	7.381 (10), 0.689
	Applied Medical Sciences	118 (89.4%)	14 (10.6%)	
	Business Administration	28 (84.8%)	5 (15.2%)	
	Computer and Info Sci.	17 (89.5%)	2 (10.5%)	
	Dentistry	1 (100.0%)	0 (0.0%)	
	Education	7 (87.5%)	1 (12.5%)	
	Engineering	13 (100.0%)	0 (0.0%)	
	Nursing	8 (88.9%)	1 (11.1%)	
	Science	15 (83.3%)	3 (16.7%)	
	Medicine	6 (66.7%)	3 (33.3%)	
	Sharia and Law	8 (80.0%)	2 (20.0%)	
Academic year	1	56 (87.5%)	8 (12.5%)	2.180 (6), 0.902
	2	54 (85.7%)	9 (14.3%)	
	3	41 (89.1%)	5 (10.9%)	
	4	23 (82.1%)	5 (17.9%)	
	5	39 (92.9%)	3 (7.1%)	
	6	6 (85.7%)	1 (14.3%)	
	7	6 (85.7%)	1 (14.3%)	
Educational level	Bachelor’s	205 (88.7%)	26 (11.3%)	2.996 (1), 0.083
	Master’s	20 (76.9%)	6 (23.1%)	
Academic load	Low	23 (82.1%)	5 (17.9%)	3.594 (3), 0.309
	Moderate	141 (86.0%)	23 (14.0%)	
	High	39 (92.9%)	3 (7.1%)	
	Very high	22 (95.7%)	1 (4.3%)	
Smoking status	Non-smoker	212 (88.7%)	27 (11.3%)	4.171 (1), 0.041
	Smoker	13 (72.2%)	5 (27.8%)	
Consulted doctor for chronic MSP	No	189 (85.5%)	32 (14.5%)	5.954 (1), 0.015
	Yes	36 (100.0%)	0 (0.0%)	
Mobile-phone use (h/day)	1–2	2 (100.0%)	0 (0.0%)	1.640 (4), 0.802
	3–4	44 (89.8%)	5 (10.2%)	
	5–6	84 (89.4%)	10 (10.6%)	
	7–9	64 (84.2%)	12 (15.8%)	
	≥10	31 (86.1%)	5 (13.9%)	
Desktop/iPad for study (h/day)	1–2	55 (88.7%)	7 (11.3%)	1.952 (4), 0.745
	3–4	86 (84.3%)	16 (15.7%)	
	5–6	45 (91.8%)	4 (8.2%)	
	7–9	30 (88.2%)	4 (11.8%)	
	≥10	9 (90.0%)	1 (10.0%)	
Breaks during study	Rarely	71 (92.2%)	6 (7.8%)	3.314 (2), 0.191
	≤30 min	61 (82.4%)	13 (17.6%)	
	≥1 h	129 (87.2%)	19 (12.8%)	
Study posture	Desk + chair	55 (88.7%)	7 (11.3%)	0.048 (1), 0.827
	Floor	96 (88.1%)	13 (11.9%)	
	Sofa/bed	19 (100.0%)	0 (0.0%)	
Sleep hours/night	1–2	189 (100.0%)	0 (0.0%)	6.038 (3), 0.110
	3–4	36 (100.0%)	0 (0.0%)	
	5–6	2 (100.0%)	0 (0.0%)	
	7–9	44 (89.8%)	5 (10.2%)	
Sleeping position	Side	8 (100.0%)	0 (0.0%)	1.075 (2), 0.584
	Back	16 (88.9%)	2 (11.1%)	
	Tummy	1 (0.0%)	0 (0.0%)	
Sleep discomfort (position change)	No	158 (86.4%)	25 (13.6%)	5.793 (1), 0.016
	Yes	26 (92.9%)	2 (7.1%)	
Bedtime	10–11 PM	66 (86.8%)	10 (13.2%)	14.162 (12), 0.290
	11–12 PM	109 (87.9%)	15 (12.1%)	
	12–1 AM	29 (82.9%)	6 (17.1%)	
	1–3 AM	21 (95.5%)	1 (4.5%)	
	8–9 PM	53 (98.1%)	1 (1.9%)	
	9–10 PM	50 (75.8%)	16 (24.2%)	
	After 2 AM	3 (75.0%)	1 (25.0%)	
Physical activity type	Aerobic	66 (86.8%)	10 (13.2%)	2.017 (3), 0.569
	None	109 (87.9%)	15 (12.1%)	
	Resistance	29 (82.9%)	6 (17.1%)	
	Stretching/Yoga	21 (95.5%)	1 (4.5%)	
Perceived stress level	Low	50 (75.8%)	16 (24.2%)	14.267 (2), 0.001 *
	Moderate	122 (89.1%)	15 (10.9%)	
	High	53 (98.1%)	1 (1.9%)	
IPAQ activity category	High	3 (75.0%)	1 (25.0%)	0.587 (2), 0.746
	Low	36 (87.8%)	5 (12.2%)	
	Moderate	186 (87.7%)	26 (12.3%)	

Note. Values are counts with row percentages in parentheses. Percentages are calculated from the raw *n* values and displayed to one decimal place. Pearson’s χ^2^ test with two-tailed significance was used for all comparisons; degrees of freedom (df) appear in parentheses after each χ^2^ value. Boldface (or asterisks) may be used to denote statistical significance at *p* < 0.05. MSP = musculoskeletal pain, IPAQ = International Physical Activity Questionnaire.

**Table 3:** Multivariate logistic regression predicting 12-month musculoskeletal pain presence (N = 257).

Predictor	Wald χ^2^	*p* Value	Adjusted OR (Exp β)	95% CI for OR
Intercept	1.791	0.181	10.247	—
Sex (male)	1.21	0.271	0.597	0.239–1.496
Perceived Stress Scale total (per point)	10.732	0.001	1.087	1.034–1.143
Current smoker (yes)	0.774	0.379	1.843	0.472–7.190
Academic workload (very high)	2.491	0.477	—	—
Low	0.43	0.512	0.462	0.046–4.641
Moderate	0.216	0.642	0.602	0.071–5.102
High	0.116	0.733	1.528	0.133–17.548
Hours of sleep (per hour/night)	2.332	0.127	0.615	0.329–1.148

Note: OR = odds ratio; CI = confidence interval. All the predictors were entered simultaneously into one model. Only stress was a significant predictor of musculoskeletal pain (MSP).

## Data Availability

The datasets analyzed in the current study are available from the corresponding author on reasonable request.
